# Phenolic-Rich Extracts from Avocado Fruit Residues as Functional Food Ingredients with Antioxidant and Antiproliferative Properties

**DOI:** 10.3390/biom11070977

**Published:** 2021-07-02

**Authors:** Gustavo R. Velderrain-Rodríguez, Javier Quero, Jesús Osada, Olga Martín-Belloso, María Jesús Rodríguez-Yoldi

**Affiliations:** 1 Agrotecnio Center, Department of Food Technology, University of Lleida, Av. Alcalde Rovira Roure 191, 25198 Lleida, Spain; grvelderrain@gmail.com (G.R.V.-R.); olga.martin@udl.cat (O.M.-B.); 2 Department of Pharmacology and Physiology, Forensic and Legal Medicine, Veterinary Faculty, University of Zaragoza, 50013 Zaragoza, Spain; javierquero94@gmail.com (J.Q.); josada@unizar.es (J.O.); 3 Department of Biochemistry and Molecular Cell Biology, Veterinary Faculty, University of Zaragoza, 50009 Zaragoza, Spain; 4 CIBERobn, ISCIII, IIS Aragón, IA2, 28029 Madrid, Spain

**Keywords:** polyphenols, bioactive compounds, functional Ingredients, cancer, Caco-2 cells, ROS, agroindustrial byproducts

## Abstract

In this study, the total phenolic compounds content and profile, the nutritional value, the antioxidant and antiproliferative activities of avocado peel, seed coat, and seed extracts were characterized. Additionally, an in-silico analysis was performed to identify the phenolic compounds with the highest intestinal absorption and Caco-2 permeability. The avocado peel extract possessed the highest content of phenolic compounds (309.95 ± 25.33 mMol GA/100 g of extract) and the lowest effective concentration (EC_50_) against DPPH and ABTS radicals (72.64 ± 10.70 and 181.68 ± 18.47, respectively). On the other hand, the peel and seed coat extracts had the lowest energy densities (226.06 ± 0.06 kcal/100 g and 219.62 ± 0.49 kcal/100 g, respectively). Regarding the antiproliferative activity, the avocado peel extract (180 ± 40 µg/mL) showed the lowest inhibitory concentration (IC_50_), followed by the seed (200 ± 21 µg/mL) and seed coat (340 ± 32 µg/mL) extracts. The IC_50_ of the extracts induced apoptosis in Caco-2 cells at the early and late stages. According to the in-silico analysis, these results could be related to the higher Caco-2 permeability to hydroxysalidroside, salidroside, sakuranetin, and luteolin. Therefore, this study provides new insights regarding the potential use of these extracts as functional ingredients with antioxidant and antiproliferative properties and as medicinal agents in diseases related to oxidative stress such as cancer.

## 1. Introduction

Avocado peel and seed are the main agro-industrial residues generated during the avocado commercial processing and represent up to 21–30% of the total fruit weight. These residues have been identified as a rich source of bioactive compounds such as carotenoids, tocopherols, and phenolic compounds, being the latter the most abundant [1]. The main phenolic compounds identified in the avocado peel and seed are derivatives of chlorogenic acid (such as caffeoylquinic acids, and coumaroylquinic acids) and some flavonoids (such as catechins, quercetin glycosides, procyanidins) [2]. Moreover, the concentration of these phenolic compounds is far higher in the avocado peel and seed than that observed in its pulp [3]. Thus, these are residues that could be a low-cost source to obtain a wide variety of phenolic acids and flavonoids within extracts with a high functional potential. The use of these residues serves several purposes beyond the economic benefits of the food industry. The use of avocado residues aims to ease the current climatic and environmental situations, heading to a new and improved sustainable production process and, at the same time, taking advantage of the nutritional content of these residues for developing healthier food products.

In that sense, the avocado peel, seed coat, and seed extracts have been recently studied as potential ingredients to be added in novel functional food products [1,4,5]. Among these, the avocado peel has a higher phenolic compounds content than the seed, and presumably also higher than the seed coat. Even when the studies about avocado seed coat are still scarce, it has been recently reported that its phenolic compound content is comparable to that from seeds, with only some phenolic acids and flavonoids found in higher concentrations [6]. Nevertheless, all these residues (peel, seed coat and seed) contain important amounts of proanthocyanidins, catechins, and quercetin glycosides, which have been related to several health-related properties [2,7]. A regular consumption of foods rich in this type of phenolic compounds has been associated with health-related benefits due to their antioxidant, antiproliferative, anti-inflammatory, and anti-mutagenic properties [3,8].

So far, scarce studies have suggested that avocado residues might be used to obtain phenolic-rich extracts with antiproliferative properties and a potential role in cancer prevention/treatment. Regarding avocado peel extracts, it has been reported that they may induce apoptosis in MDA-MB-231 cells due to increase activation of caspase 3 and caspase 3 target protein PARP [9]. On the other hand, the avocado seed extracts have shown anti-inflammatory and antiproliferative activities against the HCT-116 (colorectal carcinoma) and the HepG-2 (liver cancer) cell lines in a dose-dependent manner [3]. As for seed coat extracts, no published studies evaluating the effect of this extract on human cancer cells are available yet. However, avocado seed coat extracts have an important amount of procyanidin pentamers, which have been proposed as strategies against pathogens producing an inflammatory response in the gastrointestinal tract, such as *Helicobacter pylori* (*H. pylori*) [6,10]. Furthermore, the polymeric forms of procyanidins have been also reported to have antioxidant effects, anti-inflammatory effects, cardiovascular system amelioration effects, and hypertension diminution effects [11]. Thus, the current scientific evidence suggests that extracts from avocado residues could be used as functional ingredients to be added in nutraceuticals or novel food products. Nevertheless, further studies are still needed to confirm the potential health-related benefits of the extracts obtained from avocado residues and their use in food products.

Thus, this work aimed at evaluating the avocado fruit residues (peel, seed coat, and seed) as potential functional ingredients to be used either in nutraceuticals or food products for the prevention and treatment of gastrointestinal diseases. In that sense, the extracts obtained from the avocado peel, seed coat, and seed were characterized in terms of phenolic compounds content and profile, macronutrient content, energy value and in-vitro antioxidant activity. In addition, the phenolic compounds profile of the avocado residues was used to evaluate their individual intestinal absorption and the Caco-2 permeability by performing an in-silico analysis. Moreover, the antiproliferative activity, and its mechanism of action, of these extracts were evaluated using undifferentiated colon cells (Caco-2). As far as we know, this is the first time that the antioxidant activity, the nutritional value, and antiproliferative properties of the peel, seed coat and seed extracts are characterized in-depth, directly compared, and tested against human cancer cells. 

## 2. Materials and Methods

Avocado fruit were purchased from a local market in Lleida, Spain. Ethanol and other solvents were purchased from Fischer Scientific (Leicestershire, UK) and Scharlau S.L. (Barcelona, Spain).

### 2.1. Avocado Peel, Seed Coat and Seed Extracts

The avocado peel, seed coat and seed were obtained from previously washed avocado fruit, as described by Velderrain-Rodríguez, Salvia-Trujillo, González-Aguilar and Martín-Belloso [1]. These residues were separated, washed, and subsequently freeze-dried before the extraction process. Briefly, the residues obtained after freeze-drying were homogenized using a kitchen blender to obtain the peel, seed, and seed coat powders. These dried powders were used to obtain the phenolic-rich extracts by maceration with an aqueous 80% ethanol solution, incubated for 20 h at 40 °C using an orbital shaker, and subsequently centrifuged at 5000 rpm for 10 min at 4 °C. Then, the extracts were filtered, and the ethanol was removed using a rotary evaporator under vacuum at 40 °C, and lastly lyophilized using a laboratory freeze-drier (Telstar Cryodos, Terrassa, Spain), and powdered prior storage.

### 2.2. Chemical Characterization of the Extracts

The chemical characterization of avocado peel, seed coat and seed extracts were assessed by total phenolic compounds, flavonoids, and anthocyanins. Firstly, the total phenolic compounds content of the extracts was determined by Folin-Ciocalteu method as described by Mazzucotelli, et al. [12]. Briefly, 20 µL of extract, 150 µL of 2N Folin-Ciocalteu reagent and 130 µL of a 7.5% sodium carbonate solution were added to a 96-well plate, incubated for 30 min and read at 765 nm using a microplate reader (Multiskan GO Microplate Spectrophotometer, Thermo Fisher Scientific, Waltham, MA, USA). A calibration curve using gallic acid (0.01–0.2 mg/mL) was used to quantify the total phenolic compounds content, and the results were expressed as mM of gallic acid equivalents per gram of dried extract (mM GA eq/100 g of extract).

Secondly, the total flavonoids content was obtained by the aluminum chloride (AlCl_3_) colorimetric method as described by Velderrain-Rodríguez, Salvia-Trujillo, González-Aguilar and Martín-Belloso [1]. An aliquot (100 µL) of extract was added to 430 µL of solution A (1.8 mL of a 5% NaNO_2_ solution with 24 mL of distilled water) and incubated for 5 min. Then, 30 µL of a 10% anhydrous AlCl_3_ solution was added to the mixture and incubated for 1 min. Later, 440 µL of solution B (12 mL of 1M NaOH with 14.4 mL of distilled water) were added to the mixture. Finally, 300 µL of the final mixture were placed into a 96-well plate, and the absorbance was read at 496 nm microplate reader (Multiskan GO Microplate Spectrophotometer). The total flavonoids content was calculated from a catechin (0.01–2.5 mg/mL) calibration curve and expressed as mM of catechin equivalents per gram of dried extract (mM C eq/100 g of extract).

Lastly, the total anthocyanins content was displayed by pH-differential method as described by Teixé-Roig, et al. [13]. An aliquot of extract was diluted using two different buffer systems, 0.025 M potassium chloride (pH 1.0) and 0.4 M sodium acetate (pH 4.5), at a 1:3 (sample:solvent) ratio. Then, the absorbance was measured using a CECIL 2021 UV/VIS spectrophotometer (Cecil Instruments, Cambridge, UK) at 515 and 700 nm, against distilled water as blank. The total anthocyanin (TA) content (mg/L) was quantified according to Equation (1):

(1) $$TA=\frac{\left[\left[{\left(A_{515}-A_{700}\right)}_{pH\;1.0}-{\left(A_{515}-A_{700}\right)}_{pH\;4.5}\right]\;✕\;MW\;✕\;DF\;✕\;1000\right]}{\varepsilon \;✕\;L}$$

where *MW* is the molecular weight of cyanidin-3-glucoside (449.2 g/mol), *DF* is the dilution factor, L is the path length (cm), and ε is the molar extinction coefficient for cyanidin-3-glucoside (26,900 L mol^−1^ cm^−1^). Results were expressed as mMol cyanidin-3-glucoside equivalents per 100 g of dried extract (mMol Cyanidin-3-gucoside eq/100 g of extract).

### 2.3. Determination of Antioxidant Activity of Avocado Extracts

The antioxidant activity of the avocado peel, seed coat and seed extracts was measured by three methods: 2,2-diphenyl-1-picrylhydrazyl (DPPH), 2,2′-azinobis(3-ethylbenzothiaziline-6-sulfonate) (ABTS) and ferric reducing antioxidant power (FRAP), adapted to a microplate reader as performed by Velderrain-Rodríguez, Salvia-Trujillo, González-Aguilar and Martín-Belloso [1]. The scavenging activity for DPPH and ABTS radical was evaluated using different concentrations of the avocado peel, sed coat and seed extracts (0, 15, 30, 60, 125 and 250 µg/mL). Results were expressed by either the µMol Trolox equivalents per 100 g of extract (µMol Trolox eq/100 g of extract) and the effective concentration (EC_50_) of extract required to scavenge 50 percent of initial concentration of the free radical generated. The EC_50_ was obtained from a linear regression plot of percentage inhibition against the extract concentration (µg/mL). As for the FRAP assay, the results were expressed as µMol Trolox equivalents per 100 g of extract (µMol Trolox eq/100 g of extract).

### 2.4. Profile of Phenolic Compounds in the Extracts 

The identification and quantification of the phenolic compounds in the avocado peel, seed coat and seed extracts was performed as described by López-Gámez, et al. [14]. Thus, the phenolic compound profile in these extracts was obtained using an AcQuity Ultra-Performance™ liquid chromatography (UPLC) coupled to a triple quadrupole detector (TQD) mass spectrometer (Waters, Milford). The analytical column was an AcQuity BEH C_18_ column (100 mm × 2.1 mm i.d., 1.7 μm,) equipped with a VanGuard™ Pre-Column AcQuity BEH C_18_ (2.1 × 5 mm; 1.7 μm), also from Waters. During the analysis, the column was kept at 30 °C, and the flow rate was 0.3 mL min^−1^. Tandem MS analyses were performed using a triple quadrupole detector (TQD) mass spectrometer (Waters, Milford, MA, USA) equipped with a Z-spray electrospray interface (ESI). The mass spectrometry was operated in negative mode ESI, acquiring the data through selected reaction monitoring (SRM). Two SRM transitions were selected, the most sensitive transition for quantification and a second one for confirmation purposes. The dwell time established for each transition was 30 ms. The phenolic compounds standards used for the identification and quantification are listed in the Supplementary Table S1. The MW, SRM for quantification, cone voltages and collision energies for each compound are listed in the Supplementary Table S2. Data acquisition was carried out with the MassLynx 4.1 software. Results were expressed on a dry weight basis as µg of individual phenolic compound per 100 g of extract (µg/100 g of extract).

### 2.5. Macronutrients and Energy Content of Extract

The macronutrients content in the avocado peel, seed coat and seed extracts were determined in terms of the total carbohydrates, protein, and fat content. The total carbohydrates were determined using the anthrone method as described by Enneb, et al. [15]. The total carbohydrates content in the extracts was calculated using a calibration curve of glucose and expressed as percentage of carbohydrates in the extracts. The total protein content was determined in the extracts was obtained by Bradford colorimetric assay, as described by Enneb, Drine, Bagues, Triki, Boussora, Guasmi, Nagaz and Ferchichi [15]. The total protein content was calculated using a calibration curve of bovine serum albumin and expressed as percentage of protein in the extracts. As for the fat content, it was determined using Bligh and Dyer method for total lipids analysis as described by Chua, et al. [16]. The total fat content was determined gravimetrically and expressed as percentage of fat in the extracts. Moreover, the moisture and ash quantification was also performed as described by Ferreira, et al. [17], determined gravimetrically and expressed as percentage in the extracts. Lastly, the energy value of each extract was determined as described by Demoliner, et al. [18] using the Equation (2):

(2) $$Total\;energy\;\left(\mathrm{kcal}/100\;g\right)=4\;✕\;(g\;carbohydrates\;+\;g\;proteins)+9\;✕\;(g\;Fat)$$

### 2.6. Cell Culture, Cell Treatment and Determination of Citotoxicity

Human Caco-2 cell line (TC7 clone) was kindly provided by Dr. Edith Brot-Laroche (Université Pierre et Marie Curie-Paris 6, UMR S 872, Les Cordeliers, France). The cells were maintained in a humidified atmosphere of 5% CO_2_ at 37 °C. Cells were grown in Dulbecco’s Modified Eagles medium (DMEM) (Gibco Invitrogen, Paisley, UK) supplemented with 20% fetal bovine serum (FBS), 1% non-essential amino acids, 1% penicillin (1000 U/mL), 1% streptomycin (1000 μg/mL) and 1% amphotericin (250 U/mL). The cells were passaged enzymatically with 0.25% trypsin-1 mM EDTA and sub-cultured on 25 cm^2^ plastic flasks at a density of 5 × 10^5^ cells/cm^2^. Culture medium was replaced every 2 days. Extract treatments were added 24 h post-seeding for assays on Caco-2 undifferentiated cells (Garcia-Moreno et al., 2013); and 10–15 days post-seeding on differentiated Caco-2. Cell confluence (80%) was confirmed by optical microscopy. The choice of these cells was related to the possibility of working with the same cell line in undifferentiated cancer cells and normal enterocytes.

Extracts from avocado residues (peel, seed coat and seed) were diluted in cell culture medium to the final concentration 1.2 mg/mL. A range of concentrations of the avocado extracts (62.5, 125, 250, 500, and 1000 μg/mL) was tested. These concentrations were chosen based on previous work, carried out by our research group, using different plant extracts [19,20,21]. For cytotoxicity screening assays, the cells were seeded in 96-well plates at a density of 4 × 10^3^ cells/well. Culture medium was replaced with medium containing all different avocado extracts and cells were incubated for 24, 48 or 72 h. Antiproliferative effect was measured with the fluorometric cell viability Resazurin assay [22]. Resazurin stock solution at a concentration of 10 mg/mL was prepared dissolving Resazurin powder in phosphate-buffered saline (PBS) and this stock solution was re-diluted 1:10 (with respect of total volume) in DMEM to obtain the working solution. After removing culture medium from plates, 100 µL of working solution were added to every well and cells were incubated at 37 °C for 2 h. Finally, fluorescence was measured with excitation/emission at 544/590 nm using a FLUOstar Omega (BMG Labtech) microplate reader. The effect on cell growth was expressed as a percentage of the control. Finally, the inhibitory concentration required to reduce 50% of cell viability (IC_50_) was calculated under all conditions tested. This value was selected for further analysis to elucidate their mechanism of action on cancer cells.

### 2.7. Cell Death Studies

Caco-2 cells were seeded in 25 cm^2^ flasks (5 × 10^5^ cells/cm^2^) and then exposed to avocado extracts for 72 h at IC_50_ concentration, then collected and stained with Annexin V-FITC and propidium iodide as previously described by Sanchez-de-Diego, et al. [23]. A negative control was prepared by untreated cells, that was used to define the basal level of apoptotic and necrotic or dead cells. After incubation, cells were transferred to flow cytometry tubes and washed twice with phosphate saline buffer (PBS), followed by a resuspension in 100 µL of annexing V binding buffer (100 mM Hepes/NaOH pH 7.4, 140 mM NaCl, 2.5 mM CaCl2). 5 µL annexin V-FITC and 5 µL propidium iodide were added to each tube. After 15 min of incubation at room temperature covered from light, 400 µL of annexin binding buffer were added and analyzed by flow cytometry within 1 h. The signal intensity was measured using a FACSARIA BD and analyzed using FASCDIVA BD.

### 2.8. Flow Cytometry Mitochondrial Membrane Potential Assay 

Cells were seeded in 25 cm^2^ flasks and then exposed to avocado extracts for 72 h. The control cells were incubated with a new medium without treatment. Then, cells were washed twice with PBS. The pellet was resuspended in PBS at concentration of 106 cell/mL and 5 µL of 10 µM 1,1′,3,3,3′-hexamethylindodicarbo-cyanine iodide (DiIC1) were added to each sample. Tubes were incubated at 37 °C for 15 min and 400 µL PBS were added prior to analyze fluorescence with FACSARRAY BD equipped with an argon ion laser. Excitation and emission setting were 633 and 658 nm, respectively [23]. 

### 2.9. Determination of Intracellular Levels of Reactive Oxygen Species (ROS) 

Caco-2 cells were seeded in 96-wells plate at a density of 4 × 10^3^ cells/well. The intracellular level of ROS was assessed using the dichlorofluorescein assay as previously described by Sanchez-de-Diego, Marmol, Perez, Gascon, Rodriguez-Yoldi and Cerrada [23]. Cells were cultured before oxidative stress induction, and then incubated with stem extracts for 24 h. After that, the medium was removed, cells were washed twice with phosphate buffered saline, and incubated for 1 h with 20 µM 2′,7′–dichlorofluorescein diacetate (DCFH-DA) in PBS at 37 °C. The formation of the fluorescence oxidized derivative of DCF was monitored at an emission wavelength of 535 nm and an excitation of 485 nm in a multiplate reader. A measure at time “zero” was performed, cells were then incubated at 37 °C in the multiplate reader, and generation of fluorescence was measured after 20 min. ROS levels were expressed as a percentage of fluorescence compared to the control. The obtained values of fluorescence intensity are considered as a reflection of total intracellular reactive oxygen species (ROS) content. 

### 2.10. Theoretical Absorption Percentage of Individual Phenolic Compounds

Chemical structures and SMILES (simplified molecular-input line-entry system) codes of the individual phenolic compounds identified by UPLC-ESI-MS/MS were obtained from the PubChem Open Chemistry Database (https://pubchem.ncbi.nlm.nih.gov/search/, accessed on 12 June 2021) [24]. Relevant molecular features related to their enteral absorption capacity [molecular weight (MW; g/mol), total polar surface area (TPSA), octanol/water partition coefficient (LogPo/w), Lipinski’s rule of five (LIRF) and theoretical percentage of absorption (% Abs)] were further obtained by using the “Molinspiration online property calculation toolkit” (http://www.molinspiration.com/, accessed on 12 June 2021) as described by Ertl and Schuffenhauer [25]. Moreover, the online program pkCSM (http://biosig.unimelb.edu.au/pkcsm/prediction, accessed on 12 June 2021) was used to predict the Caco-2 permeability given as the logarithm of the apparent permeability coefficient (log P_app_) expressed in 10^−6^ cm/s.

### 2.11. Statistical Analysis

All assays were performed at least three times. Data are presented as mean ± standard deviation. Means were compared using one-way analysis of variance (ANOVA). For the chemical characterization, the antioxidant activity, macronutrients characterization and individual phenolic compounds content of the extracts, the differences between means were compared by the Tukey-Kramer multiple comparison test (*p* < 0.05) using the statistical software NCSS 2007. As for the results obtained from the assays using Caco-2 cells, the significant differences at *p* < 0.05 were compared using a Bonferroni’s Multiple Comparison Test using the GraphPad Prism Version 5.02 program.

## 3. Results and Discussion

### 3.1. Chemical Composition and Antioxidant Activity

The chemical characterization of the avocado’s peel, seed coat, and seed extracts was addressed in terms of total phenolics, flavonoids, and anthocyanins. In all cases, the avocado peel extracts showed a higher content of phenolic species compared to that observed in the seed coat and seed extracts, as it is shown in Table 1. According to these results, no statistical differences were observed between the seed coat and seed extracts, with a lower total phenolic content than that observed for the avocado peel extracts (309.95 ± 25.33 mMol GA/100 g of extract). As for the total flavonoids content, the peel extracts showed a 3.6- and 5.8-fold higher total flavonoid content compared to the seed coat and seed extracts, respectively. Regarding anthocyanins content, the avocado peel extracts also had the highest content, showing a 1.08- and 1.72-fold higher total anthocyanins content compared to the seed coat and seed, respectively. To the best of our knowledge, there are no studies directly comparing the phenolic compounds from avocado seed coat extract to those obtained from the peel. However, the comparison between the peel and seed extracts has been recently addressed and has reported a higher content of bioactive compounds in avocado peel extracts compared to that from its seed [1,26]. Moreover, Figueroa, Borrás-Linares, Lozano-Sánchez and Segura-Carretero [6] reported that the avocado seed coat extracts had a higher content of flavonoids, some phenolic acids and organic acids compared to that observed in the seed extracts.

The phenolic compounds have been related to a diverse pharmaceutical and health-promoting effects, mostly due to their antioxidant and anti-inflammatory properties. These properties are highly dependent of the different molecular structures of the phenolic species, as these are related to their ability to donate hydrogen or electrons to free radicals [27]. In that sense, phenolic compounds are considered as primary antioxidants, due to their ability to neutralize free radicals either by donating an H-atom (hydrogen atom transfer, abbreviated as HAT) or by a single electron transfer (SET) mechanism. Among the different types of phenolic compounds, it has been demonstrated that the number and arrangement of their hydroxyl groups are responsible for their different antioxidant activity values [28]. Therefore, the characterization of the total phenolic compounds within the avocado peel, seed coat, and seed extracts used in this study was the first step needed to picture the potential beneficial effects of these residues.

In consequence, the next step was to evaluate the antioxidant properties of the avocado’s peel, seed coat, and seed extracts by DPPH, ABTS, and FRAP assays. Different antioxidant assays were performed to evaluate the occurrence of different antioxidant/reaction mechanisms (HAT or SET) followed by the radicals and the extracts. Specifically, DPPH and ABTS assays react by a mixed HAT and SET mechanism, whereas FRAP assay reacts solely through the SET mechanism. As it is shown in Table 2, results from the DPPH assay indicates that the antioxidant activity of the avocado peel extracts (46.49 ± 4.04 µMol Trolox eq./100 g of extract) was significantly higher to that observed in the seed coat (36.80 ± 11.03 µMol Trolox eq./100 g of extract) and seed (32.51 ± 9.07 µMol Trolox eq./100 g of extract) extracts. Moreover, no statistical differences were observed between the antioxidant activity of the seed coat and seed extracts. In addition, these results also suggest that even when the peel extract had a higher antioxidant activity value against DPPH radical, no differences were observed between the half-maximal inhibitory concentration (EC_50_) of the peel (72.64 ± 10.70 µg/mL) and seed coat (82.13 ± 2.54 µg/mL) extracts. As for the antioxidant activity against the ABTS radical, the peel extracts also had higher values compared to those observed for the seed coat and seed extracts. Similarly, the lowest IC_50_ was observed in the peel extract (181.68 ± 18.47 µg/mL), followed by the seed coat (260.29 ± 16.41 µg/mL) and seed (318.68 µg/mL) extracts. Thus, according to these results, the peel and seed coat extracts are more effective scavengers against DPPH and ABTS radicals, compared to seed extracts. Interestingly, the results obtained from FRAP assay shown that the seed coat extracts had a 1.7- and 2.6-fold higher antioxidant activity value compared to that observed for peel and seed extracts. The higher antioxidant activity of the seed coat extracts observed in FRAP assay, compared to that of peel and seed extracts, suggests the presence of a higher number of phenolic species following the SET antioxidant mechanism [29].

Therefore, the higher antioxidant values found in the avocado peel extract could be related to its higher content of phenolic compounds. The superiority of the avocado peel extracts, in terms of phenolic compounds content, compared to those from pulp and seed has also been reported in other studies [1,26]. Moreover, a recent study performed by Figueroa, Borrás-Linares, Lozano-Sánchez and Segura-Carretero [6] reported that the avocado seed coat extract has a higher content of flavonoids, such as catechin, compared to the seed extract. Catechins has been reported to be molecules with a high antioxidant activity and an efficient SET capacity [30]. This could explain the higher antioxidant value of the avocado seed coat extracts observed in the FRAP assay. Moreover, it is known that the antioxidant activity of anthocyanins is higher than those of phenolic acids and some flavonoids. Therefore, the higher content of anthocyanins in avocado peel and seed coat extracts may be related to its higher antioxidant activity.

### 3.2. Quantification of the Individual Phenolic Compounds in Avocado Peel, Seed Coat and Seed Extracts by UPLC-ESI-MS/MS

Measuring concentrations of total phenolics, flavonoids and anthocyanins in the avocado peel, seed coat and seed extracts using UV/Vis spectrophotometry offers a rapid chemical index, but chromatographic techniques are necessary to establish structure-activity evidences. In that sense, an identification and quantification of phenolic compounds in the avocado peel, seed coat and seed extracts by UPLC-ESI-MS/MS was performed and display in Table 3. For comparison purposes, the identified phenolic compounds were also grouped by their different chemical structures (phenolic acids, flavonoids, and terpenes) and represented either as the individual compounds, the sum of each group and the total content found in the peel, seed coat, and seed extracts. These results reassert those obtained by spectrophotometric methods performed for their chemical characterization, as the avocado peel extracts had a 1.77- and 4.51-fold higher total phenolic compounds content compared to seed coat and seed extracts, respectively. Regarding the different groups of phenolic compounds, no differences were observed between the phenolic acids content in the peel and seed coat extracts, having both a significantly higher content than that observed for the seed extract. Regarding the total flavonoids, the highest content was observed in the peel extract, whereas no significant differences were observed between the seed coat and seed extracts. Lastly, similar results were observed for the terpenes content, with the highest content in the peel extract, followed by seed coat and seed extracts.

In addition, 72 different compounds were identified in the avocado peel, seed coat, and seed extracts. A higher diversity of compounds was observed in the peel extract, as it had 69 different phenolic compounds identified, followed by both the seed coat’ and seed’ extracts with 58 different compounds each. Furthermore, as it was mentioned previously, even when there were no differences between the total phenolic acids content of the peel and seed coat extracts, the latter had a significantly higher content of trans-cinnamic acids derivative molecules, such as the esters formed between either caffeic or ferulic acid, and quinic acid. Nevertheless, the phenolic acids with the higher content in the seed extracts were tyrosol-derived phenolic molecules, such as salidroside and hydroxysalidroside. Among flavonoids, those found in higher concentration within the avocado peel extract were epicatechin and the type B procyanidin dimers. The type B procyanidin dimers and trimers were also between the most abundant flavonoids in the seed coat extracts, along with catechin, epicatechin, and type A procyanidin dimers and trimers. Regarding seed extract, sakuranetin and luteolin were the only two flavonoids found in a higher concentration than that observed for the peel and seed coat extracts. As for terpenes, penstemide was the only found in the avocado peel, seed coat and seed extracts.

Caffeoylquinic and Feruoylquinic acid derivatives were among the most abundant phenolic acids found in the avocado peel, seed coat, and seed extracts. Along with coumaroyl- and dicaffeoyl- quinic acids, these molecules are considered as isomeric forms of chlorogenic acid [31]. The chlorogenic acids play an important role as bioactive compounds with pharmacological effects such as antioxidants, free radical scavengers, and a central nervous system stimulator [32]. Moreover, catechin and epicatechin derivatives, such as procyanidins monomeric and oligomeric were the most representative flavonoids in the three extracts used in this study. Procyanidins are suggested to exert physiological and cellular activities that promote homeostasis [33]. The two main types of procyanidin oligomers found in plant-based foods are the type A and type B procyanidin oligomers. On the one hand, the type-A procyanidins oligomers have two linkages, which include a C4–C8 bond and an additional ether bond, whereas the type B procyanidin oligomers contain flavan-3-ol units that singly link through C4 → C8 and/or C4 → C6 bonds [34]. Lastly, penstemide, the only terpene found in the avocado peel, seed coat and seed extracts, is an isovaleroyl type iridoid glucoside uniquely present in avocado plants. Iridoids have been related to diverse health-promoting properties alleviating inflammation, depression, hyperglycemia, and thrombus, as well as lipopolysaccharide-induced apoptotic liver damage [35].

### 3.3. Macronutrients and Energy Value of Extracts

The macronutrient content and energy value of the avocado peel, seed coat, and seed extracts were evaluated in terms of carbohydrates, lipids, and proteins. Additionally, the moisture and ash content were also evaluated. According to the results shown in Table 4, the highest carbohydrate content was observed in the peel and seed extracts compared to that contained in the seed coat extract. Regarding total protein content, the peel extract had a higher content than the seed coat and seed extracts, with no significant differences between these two extracts. As for the total lipids, the highest content was observed in the seed extract, followed by the seed coat and peel extracts. Conversely, seed extracts had the lowest moisture content compared to the peel and seed coat extracts, which had no statistical differences between them. Moreover, the highest ash content was observed in the peel extract, followed by the seed coat and seed extracts. Lastly, the highest energy value was observed in the seed extract, whereas no statistical differences could be observed between the peel and seed coat extracts. These results show that these extracts have a great potential for being used as a source of the ingredients of high nutritional value and especially phenolic compounds. As far as we know, there are no previous reports on the nutritional characterization of extracts from these avocado residues.

The macronutrients composition and the energy value of phenolic rich extracts obtained from agroindustrial residues still scarce [36,37]. Compared to the extract obtained by Costa, et al. [38] the avocado peel extract have a similar composition of carbohydrates, proteins, and fats, whereas those from the seed coat and seed have a higher energy value and a significantly higher fat content. In addition, compared to other agro-industrial residues, such as the seed of cupuassu (a fruit from the Brazilian Amazon), the avocado seed extract had similar carbohydrates and fats content, but a lower energy value [39]. These results suggest that these extracts are not only a great source of phenolic compounds, but also contain an important concentration of macronutrients compared to either other extracts or agro-industrial residues reported in other studies. 

### 3.4. Effect of Peel, Seed Coat and Seed Avocado Extracts on Colonorectal Cancer Cells (Caco-2 Cells)

So far, the scarce studies on avocado fruit and its residues has shown that there is a potential within the food and pharmaceutical industries, as these residues have a high content of phenolic compounds and nutritional value [40]. Several studies have shown that the extracts from the avocado pulp can selectively inhibit growth and increase apoptosis in some cancer cell lines [41,42,43,44]. For instance, it has been reported that extracts from the avocado pulp can inhibit the growth of some colon cancer cells lines (HCT-116 and HTC-29) [45,46]. However, little attention has been given to the antiproliferative properties of the extracts from the avocado residues, such as avocado peel, seed coat and seed. Thus, further studies using on a human cell model to evaluate the antiproliferative properties of these extracts are still needed. In this work, the effect of the extracts from avocado residues (peel, seed coat and seed) on human colon adenocarcinoma cells (Caco-2) was evaluated in terms of antiproliferative activity, apoptosis analysis, ROS intracellular levels and the cellular antioxidant activity.

#### 3.4.1. Antiproliferative Activity

**Figure 1:**
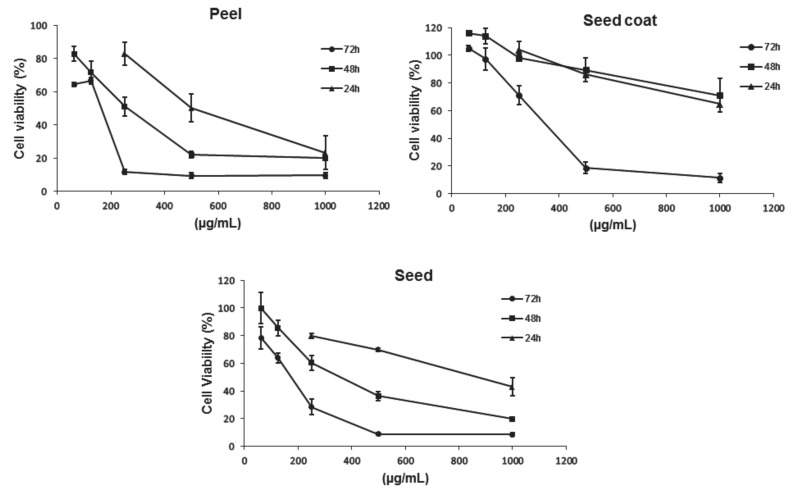
Measurement of cell viability in Caco-2 cells after incubation with peel, seed coat and seed avocado extracts at 62.5, 125, 250, 500 and 1000 μg/mL for 48 and 72 h. The concentrations 62.5 and 125 μg/mL were suppressed at the incubation time of 24 h.

The results showed in Figure 1 indicated that the avocado peel, seed coat and seed extracts decreased the viability of Caco-2 cells in a dose- and time-dependent manner. According to these results, the avocado peel and seed extracts produced a greater antiproliferative effect than the seed coat extracts as shown by their lower IC_50_. In that sense, the lowest IC_50_, after 72 h, was observed for the avocado peel extract (180 ± 40 µg/mL), followed by the seed (200 ± 21 µg/mL) and seed coat (340 ± 32 µg/mL) extracts. As it was previously shown in Table 1 and Table 3, a higher content of total phenolics was observed in the avocado peel and seed coat extracts, compared to that from seed. Thus, the fact that the seed extract shown a lower IC_50_ than that observed for the avocado seed coat extract suggests that the antiproliferative activity of these extracts is affected by the type of phenolic species rather than its concentration. Among phenolic acids, hydroxysalidroside and salidroside were found in the avocado seed extract in a higher concentration than that observed for the avocado seed coat. As for the flavonoids, the amount of sakuranetin and luteolin was significantly higher in the avocado seed extract than in the avocado seed coat extract. In the case of the avocado peel extract, the antiproliferative activity may be related either to its higher content of phenolic compounds, or its greater content of individual phenolic compounds, such as some flavonoids and phenolic acids.

Salidroside and hydroxysalidroside have been linked to diverse health-related properties. For example, salidroside has been related to various pharmacological properties, including antiaging, anticancer, anti-inflammation, hepatoprotective, and antioxidative effects antiasthma effects [47]. As for hydroxysalidroside, Horvathova, et al. [48] reported that this molecule can protect plasmid DNA against Fe^2+^-induced damage at different concentrations. Similarly, several health-related benefits have been correlated to sakuranetin and luteolin molecules. Navarro-Salcedo, et al. [49] reported that the effectiveness of pure sakuranetin (IC_50_ = 10 to 30 µg/mL) against esophageal carcinoma EC-109 cells was comparable to that observed for the acetonitrile extract obtained from the leaves of *Artemisia dracunculus* plants. As for luteolin, diverse studies have shown that this flavonoid has strong antiproliferative activity against different human cancer cell lines, including lung cancer, myeloid leukemia, prostate cancer, and pancreatic cancer [50,51]. Therefore, the presence of these compounds may explain the differences observed in the antiproliferative activity of the avocado seed extracts and the seed coat extracts.

#### 3.4.2. Apoptosis Analysis

**Figure 2:**
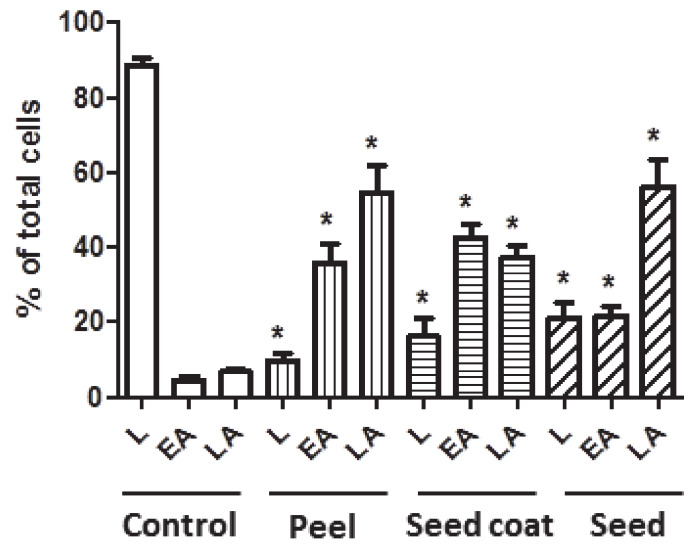
Analysis of the type of cell death induced on Caco-2 cells after 72 h incubation in Control (untreated cells) and avocado extracts at IC50 (µg/mL): peel (180), seed coat (340) and seed (200). Percentages of alive (L), early apoptotic (EA) and late apoptotic ((LA) cells are indicated. * *p* < 0.05 vs. Control (untreated cells alive, early and late apoptosis, respectively).

Even when the avocado peel, seed coat, and seed extracts reduced the viability of Caco-2 cells, the type of death and mechanisms of the action exerted by these extracts was not clear. Thus, flow cytometry analyses over 72 h were performed using biomarkers of cell death and showed in Figure 2. These results agree with those previously discussed (Figure 1) and revealed that the cells treated with the IC_50_ of each extract induced apoptosis in the early and late stages. Moreover, the most marked apoptosis effect was observed for the avocado peel extracts, which could be related to the higher concentration of phenolic compounds, as previously discussed. These results suggests that all avocado extracts at their IC_50_ induces apoptosis in Caco-2 cells by activating apoptotic pathways, thereby reducing their ability to non-selectively react with biological targets to cause necrosis and its related side effects.

According to Yu, Li, Zhao, Wang and Feng [47], salidroside induces apoptosis in human ovarian cancer SKOV3 and A2780 cells by the activation of caspase-3 and causing the upregulation levels of apoptosis-inducing factor, Bcl-2-associated X and Bcl-2-associated death promoter (Bad) proteins. Moreover, these authors stated that salidroside downregulated the levels of Bcl-2, p-Bad and X-linked inhibitor of apoptosis proteins and activated the caspase-dependent pathway in SKOV3 and A2780 cells, upregulating p53, p21Cip1/Waf1 and p16INK4a. Similarly, Jang, et al. [52] reported that luteolin can activate the Nrf2/ARE/HO-1 signaling pathway and promote p53-dependent and independent apoptotic pathways. In agreement, Tavsan and Kayali [8] reported that luteolin induced apoptosis via the activation of tumor suppressor p53, and inactivation of receptor tyrosine kinase and topoisomerases sensitization to tumor necrosis factor-α. Thus, the presence of these phenolic compounds in the extracts used in this study may explain the apoptosis effects observed in Caco-2 cells.

**Figure 3:**
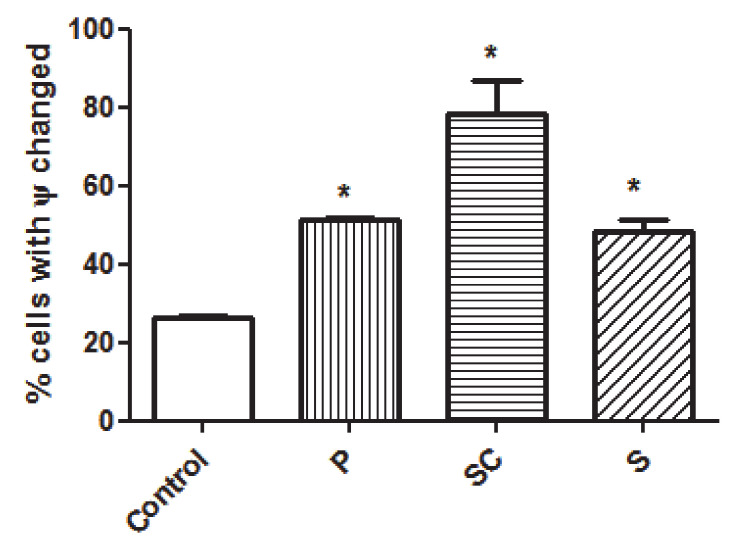
Analyses of mitochondrial membrane potential (ΔΨm) after 72 h incubation with peel (P), seed coat (SC) and seed (S) extracts at their IC50 (μg/mL) 180, 340 and 200, respectively. * *p* < 0.05 vs. Control (untreated cells).

Since previous studies on plant extracts suggested mitochondrial dysfunction and intrinsic apoptosis induction [19,20,21], the mitochondrial membrane potential change was also analyzed in this study. Mitochondria play a key role in the apoptosis induction and are associated with the changes in the mitochondrial membrane permeability. The involvement of mitochondria in cell death is generally measured by following mitochondrial membrane potential [53]. As it is shown in the results from Figure 3, all the avocado extracts significantly altered the mitochondrial potential of the Caco-2 cells compared to the untreated ones, and therefore the changes in mitochondrial potential could be related to the apoptosis observed (Figure 2). Seed coat extracts show the highest number of cells with a change in mitochondrial potential, even that could be related to a lower observed late apoptosis (Figure 3).

There are two main pathways of apoptosis in mammals: the extrinsic pathway and the mitochondrial pathway. During the latter, the most important apoptosis-promoting proteins are the P53, Bcl-2, Bax, caspase-3 and PARP. Among these proteins, the changes in mitochondrial membrane permeability are determined by the relative levels of Bcl-2 and Bax. Briefly, during the mitochondrial apoptotic pathway, the mitochondrial membrane is damaged as a result of the apoptotic effect promoted by Bax, whereas Bcl-2 inhibits apoptosis by maintaining the integrity of the mitochondrial membrane [54]. Thus, the differences between the changes of the mitochondrial potential of cells treated with seed coat extracts and those treated with peel and seed extracts could also be related to the presence of salidroside, hydroxysalidrose, sakuranetin and luteolin. For example, in a recent study, it was observed that salidroside upregulated the level of Bcl-2 and downregulate the level of Bax, having a protective effect through the mitochondrial apoptosis pathway [55]. Conversely, it has been reported that luteolin can upregulate Bax and downregulate BCL-2, increasing the apoptotic rates in breast cancer cells [56]. Nevertheless, further studies should be performed to identify the main phenolic compounds within the avocado peel, seed coat and seed extracts involved in the mitochondrial apoptotic pathway.

#### 3.4.3. Effect of Avocado Extracts on ROS Intracellular Levels

**Figure 4:**
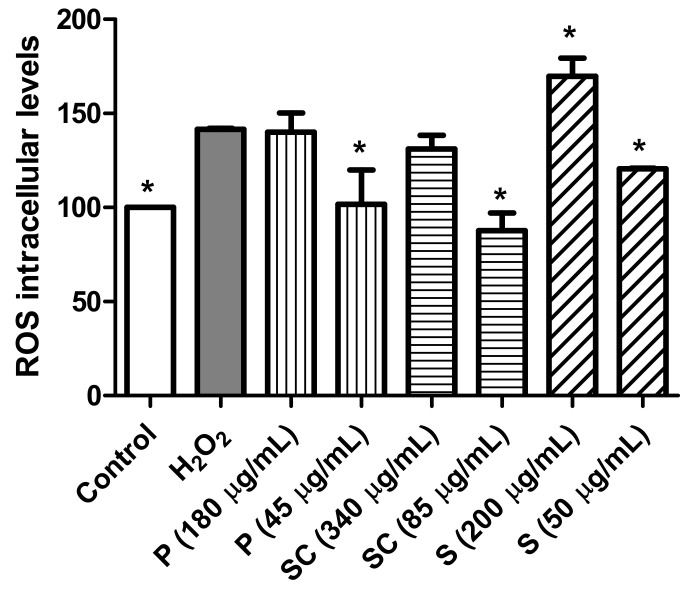
Measurements of ROS levels in presence of H2O2 (80 mM, 20 min) after 24 h incubation with peel, seed coat and seed avocado extracts at IC_50_ (180, 340 and 200 µg/mL, respectively) or 1/4 IC_50_ (45, 85 and 50 µg/mL, respectively). * *p* < 0.05 vs. H_2_O_2_.

Biochemical studies indicate that free radicals and their reactive products are responsible for chronic degenerative diseases, such as cancer. High levels of ROS are generated by increased metabolic activity of cancer cells that induce activation of signaling pathways or mitochondrial dysfunction [57]. ROS levels in the cells were determined based on the reaction between ROS and DCFH-DA. The assays were carried out by treating the cells with peel, seed coat and seed extracts in presence of hydrogen peroxide (H_2_O_2_). The H_2_O_2_ is a widespread substance used to mimic the pro-oxidative environment that characterizes degenerative diseases such as cancer or neurodegenerative disorders on 2D cell cultures. In that sense, the results from Figure 4 indicates that pro-oxidant effect in presence of hydrogen peroxide was observed in the cells treated with the avocado seed extract at a concentration of 200 µg/mL (IC_50_) and after 24 h. Furthermore, peel and seed coat extracts at their respective IC_50_ (180 and 340 µg/mL) did not show an antioxidant effect in the presence of H_2_O_2_. However, when the cells were treated with the extracts at a concentration 4 times lower than their IC_50_ (45, 85 and 50 µg/mL, respectively), a significant antioxidant effect was observed.

The antioxidant effect of phenolic compounds has been extensively studied [58] although they may also have a pro-oxidant effect. These results have been mainly observed in tumor cells and have been related to the pro-apoptotic action. The dual pro-oxidant and antioxidant behavior of phenolic plant compounds not only depends on the cell type but also on their concentration, chemical structure, and pH status [30,59]. In that sense, Ding, Han, Guo, Chin, Ding, Kinghorn and D’Ambrosio [43], found that a chloroform extract of Hass avocado extract initiates apoptosis via ROS activating in oral cancer cell line.

#### 3.4.4. Antioxidant Activity of Avocado Extracts on a Model Intestinal Barrier

The phenolic compounds have an important role in the prevention of gastrointestinal diseases related to free radicals. Considering the high antioxidant activity and the total content of phenolic compounds in the avocado extracts (Table 2) and as these extracts also showed an antioxidant effect on cancer cells at low concentrations (Figure 4), the effect at the IC_50_ concentrations obtained in undifferentiated cells on a model of the intestinal barrier (differentiated Caco-2 cells) upon exogenous oxidative stress by hydrogen peroxide insult was addressed. This cell line spontaneously acquires the phenotypic features of non-cancerous enterocytes after reaching confluence (differentiated cells). Monolayer Caco-2 cells form tight junctions and present the cylindrical polarized morphology of enterocytes, expressing functional microvilli on the apical membrane. Therefore, differentiated Caco-2 cells have been established as an acceptable in vitro intestinal barrier model. 

**Figure 5:**
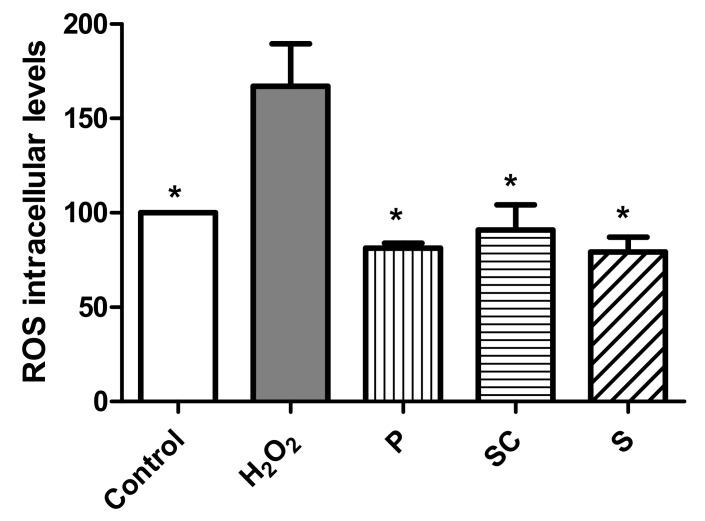
Measurements of ROS levels in presence of H_2_O_2_ (80 mM, 20 min) after 24 h incubation with peel, seed coat and seed avocado extracts at 180, 340 and 200 µg/mL, respectively. * *p* < 0.05 vs. H_2_O_2_.

In these differentiated cells, the antioxidant capacity at 24 h incubation time of the peel, seed coat and seed extracts were tested at the concentrations of 180 µg/mL, 340 µg/mL, and 200 µg/mL, respectively. The results showed a clear antioxidant effect by preventing H_2_O_2_-induced ROS production (Figure 5). In this way, plant extracts have been investigated for their capacity to correct the aberrant increase in ROS levels derived from H_2_O_2_ exogenous addition [21].

The antioxidant activity of plant extracts is strongly correlated with its clinical application in gastrointestinal diseases related to oxidative stress [60,61]. These results obtained with avocado extracts suggest that they could have a potential application in the management of gastrointestinal diseases related to oxidative stress.

#### 3.4.5. Theoretical Absorption Percentage of Individual Phenolic Compounds (Based on Lipinski Parameters)

Phenolic compounds are considered as xenobiotics, and as such, they are subjected to the same absorption, distribution, metabolism, and excretion (ADME) processes as drugs [62]. In that sense, Lipinski’s rule of five (LIRF) is used to assess the potential of drugs or xenobiotic compounds, considering the pharmacological and physiological properties needed to make them an orally active drug candidate for humans. This rule depends on simple physiochemical parameters of five, including the MW of <500 g/mol, lipophilicity (LogP) of <5, and the number of hydrogen bond donors and acceptors of <5 and <10, respectively. These parameters are associated with intestinal permeability and aqueous solubility, determining the first step of oral bioavailability [63]. In that sense, an in-silico study of the phenolic compounds identified in the avocado peel, seed coat, and seed extracts was performed by the determination of Lipinski’s parameters, topological polar surface area, and the theoretical percentage of absorption and displayed in Table 5. According to these results, the aglycone form of the phenolic acids had the highest absorption and no violations to LIRF, with values between around 80% and 90%. However, among these aglycone forms, salidroside and hydroxysalidroside had lower absorption percentages, with 67.73% and 60.76%. As for the flavonoids, one or more violations to LIRF and values below the 70% were observed for either their aglycone or glycosylated forms, except for catechin, epicatechin, naringenin, sakuranetin and luteolin. According to Hakkou, et al. [64], having two or more violations of LIRF indicates problems in the bioavailability of a standard drug or xenobiotic compound. Moreover, procyanidins had negative values, suggesting no possible absorption of these molecules. As for the terpenes, penstemide had a higher absorption percentage (82.7%) than that observed for most flavonoids, but comparable to that for sakuranetin.

The theoretical absorption percentage of the individual phenolic compounds, based on their chemical structures, may be a good indicator about their potential cellular permeability. In addition, a compound is considered to have a high Caco-2 permeability if it has a P_app_ > 8 × 10^6^ cm/s. Using the pkCSM predictive model, predicted log Papp values > 0.90 cm/s indicates a high Caco-2 permeability of the compounds tested [65]. In that sense, the P_app_ value observed for the phenolic compounds identified in the avocado peel, seed coat and seed, agrees with that absorption percentage previously discussed. Thus, the predicted data suggest that *p*-hydroxybenzoic acid, vanillin, hydroxytyrosol and *p*-coumaric acid are the phenolic acids with higher Caco-2 permeability. As for the flavonoids, those with the higher Caco-2 permeability were naringenin, sakuranetin and luteolin. Moreover, the terpene penstemide had also higher permeability values. Thus, the results of this study suggest that the antiproliferative activity of the peel, seed coat and seed extracts observed in this study may be related to the higher absorption and permeability values of these compounds.

## 4. Conclusions

This work proved that avocado residues are an important source of phenolic compounds and macronutrients that could be used in the development of novel food products. Moreover, this study highlighted the value of the extracts obtained from avocado residues to be used as promising sources for functional food or nutraceutical products with antioxidant and antiproliferative properties. In that sense, the peel, seed coat, and seed extracts have shown potent cytotoxic activity in colon cancer cells and protective effect in the intestinal barrier. The highest content of phenolic compounds and antioxidant activity values was found in the avocado peel extracts, followed by seed coat extracts. As for the nutritional value of these extracts, both peel and seed coat extracts had the lowest energy value compared to seed extracts. Nevertheless, all the avocado extracts used in this study showed, in cancer Caco-2 cells, an antiproliferative effect mediated by apoptosis with modification of the mitochondrial potential and antioxidant effect which could have associated with a reduced risk of illnesses related to marked oxidative state like cancer. In addition, all the extracts showed an antioxidant effect on differentiated intestinal cells which could protect the intestine from diseases related to oxidative stress. According to the in-silico analysis, the higher antiproliferative effect observed in avocado peel and seed extracts, compared to that from seed coat, could be related to the higher absorption and permeability of some phenolic acids, such as hydroxysalidroside and salidroside, or flavonoids such as sakuranetin and luteolin. However, the effect of these individual compounds should be evaluated in further studies to elucidate the exact role and contribution to human health. Therefore, these avocado extracts are expected to be a natural source of bioactive compounds used for the development of functional food and medical agents to prevent or treat human colon cancer.

## Figures and Tables

**Table 1:** Chemical characterization of the avocado’s peel, seed coat and seed total phenolic compounds, flavonoids and anthocyanins content.

	PEEL	SEED COAT	SEED
Total phenolics (mMol GA/100 g of extract)	309.95 ± 25.33 a	208.87 ± 11.67 b	232.36 ± 12.25 b
Total Flavonoids (mMol Cat. eq./100 g of extract)	12.54 ± 0.52 a	3.41 ± 0.36 b	2.13 ± 0.22 c
Total anthocyanins (mMol Cyanidin-3-glucoside eq./100 g of extract)	622.37 ± 17.26 a	575.76 ± 20.18 b	359.85 ± 12.61 c

Data are means ± standard deviation (n = 3). Different letters indicate a statistical difference (*p* < 0.05) between the peel, seed coat and seed extracts.

**Table 2:** Antioxidant activity of the peel, seed coat and seed extracts from avocado fruit.

	PEEL	SEED COAT	SEED
DPPH (mMol Trolox eq./100 g of extract)	46.49 ± 4.04 a	36.80 ± 11.03 ab	32.51 ± 9.07 b
(EC_50_ µg/mL)	72.64 ± 10.70 a	82.13 ± 2.54 ab	90.91 ± 3.59 b
ABTS (mMol Trolox eq./100 g of extract)	504.60 ± 35.50 a	391.49.68 ± 48.26 b	210.88 ± 64.11 c
(EC_50_ µg/mL)	181.68 ± 18.47 a	260.29 ± 16.41 b	318.68 ± 25.60 c
FRAP (mMol Trolox eq./100 g of extract)	379,308.80 ± 18,262.41 b	672,334.16 ± 35,505.87 a	256,338.52 ± 37,812.61 c

Data are means ± standard deviation (n = 3). Different letters indicate a statistical difference (*p* < 0.05) between the peel, seed coat, and seed extracts.

**Table 3:** Identification and quantification of phenolic compounds in the avocado peel, seed coat and seed extracts by UPLC-ESI-MS/MS (µg/100 g of extract).

No.	Phenolic Compound	Peel	Seed Coat	Seed
1	*p*-hydroxybenzoic acid	0.38 ± 0.01 a	0.19 ± 0.03 b	0.12 ± 0.00 c
2	Vanillin	0.10 ± 0.02 a	10 ± 0.10 b	0.02 ± 0.00 b
3	Vanillic acid	0.02 ± 0.00	n.d	n.d.
4	Syringic acid	0.09 ± 0.02	n.d	n.d.
5	Protocatechuic acid	2.20 ± 0.23 a	n.d	0.28 ± 0.12 b
6	Protocatechuic acid glucoside	26.10 ± 0.26 a	7.50 ± 0.12 b	1.95 ± 0.51 c
7	Hydroxytyrosol	0.18 ± 0.08 a	0.26 ± 0.00 a	0.18 ± 0.05 a
8	Hydroxytyrosol glucoside	0.13 ± 0.00 b	1.24 ± 0.08 a	0.91 ± 0.22 a
9	Hydroxysalidroside	0.101 ± 0.02 b	1.24 ± 0.20 b	5.30 ± 1.08 a
10	Hydroxytyrosol glucoside arabinoside	2.54 ± 0.10	0.02 ± 0.00	n.d.
11	Tyrosol glucoside	8.30 ± 0.52 b	51.40 ± 0.63 a	10.40 ± 2.70 b
12	Salidroside	0.53 ± 0.10 b	35.60 ± 3.84 b	148.40 ± 50.30 a
13	Tyrosol glucoside arabinoside	62.90 ± 1.09 a	1.85 ± 0.10 b	0.75 ± 0.19 b
14	*p*-coumaric acid	0.24 ± 0.011 b	0.33 ± 0.01 a	0.20 ± 0.02 c
15	Coumaric acid glucoside	0.32 ± 0.11	n.d	n.d.
16	Coumaroylquinic acid	1.38 ± 0.05 c	7.33 ± 0.03 a	3.61 ± 1.35 b
17	Caffeic acid	0.41 ± 0.11 b	2.98 ± 0.30 a	0.59 ± 0.01 b
18	Caffeic acid glucoside	0.37 ± 0.06 a	0.22 ± 0.02 b	0.09 ± 0.01 c
19	Caffeic acid glucoside derivative	0.07 ± 0.01	n.d	n.d.
20	Dihydrocaffeic acid glucoside	0.07 ± 0.00 c	0.58 ± 0.02 a	0.21 ± 0.04 b
21	Caffeoylshikimic acid	0.26 ± 0.01 b	2.11 ± 0.25 a	2210 ± 0.76 a
22	3-*O*-caffeoylquinic acid	17.60 ± 0.26 c	738.70 ± 59.80 a	176.20 ± 53.40 b
23	4-*O*-caffeoylquinic acid	9.56 ± 0.07 b	71.40 ± 7.14 a	10.70 ± 4.12 b
24	5-*O*-caffeoylquinic acid	969.20 ± 9.21 a	65.60 ± 7.81 b	11.30 ± 4.26 c
25	Dicaffeoylquinic acid	1.05 ± 0.01 a	0.02 ± 0.00 b	n.d.
26	Ferulic acid	0.25 ± 0.01 a	0.04 ± 0.01 b	0.05 ± 0.01 b
27	Ferulic acid glucoside	4.14 ± 0.11 a	3.97 ± 0.06 a	1.52 ± 0.56 b
28	Dihydroferulic acid glucoside	0.23 ± 0.01 a	0.05 ± 0.00 b	0.01 ± 0.00 c
29	4-*O*-feruoylquinic acid	0.48 ± 0.05 b	1.54 ± 0.23 a	0.22 ± 0.05 b
30	5-*O*-feruoylquinic acid	1.61 ± 0.05 a	0.59 ± 0.04 b	0.124 ± 0.06 c
31	3-*O*-feruoylquinic acid	0.00 ± 0.00 c	11.50 ± 0.66 a	2.83 ± 1.57 b
	***Phenolic acids***	***1215.17 ± 24.28 a***	***1011.57 ± 81.44 a***	***378.21 ± 121.60 b***
32	Catechin	n.d.	311.20 ± 43.10 a	280.50 ± 0.14 a
33	Epicatechin	1891.00 ± 75.70 a	571.00 ± 91.00 b	360.00 ± 140.60 b
34	Catechin glucoside	2.40 ± 0.03 b	10.80 ± 1.05 a	3.85 ± 0.08 b
35	Epicatechin glucoside	7.89 ± 0.16 a	8.61 ± 0.34 a	4.81 ± 0.66 b
36	Epigallocatechin	6.27 ± 0.28 a	1.36 ± 0.042 b	1.86 ± 0.67 b
37	Epicatechin gallate	n.d.	3.06 ± 0.11 a	1.39 ± 0.35 b
38	Catechin derivative	1.55 ± 0.32 c	5.50 ± 0.00 a	2.32 ± 0.27 b
39	Epicatechin derivative	67.30 ± 9.96 a	3.05 ± 0.01 b	1.72 ± 0.16 b
40	Procyanidin dimer (type A)	4.45 ± 0.085 c	41.00 ± 0.49 a	6.28 ± 0.88 b
41	Procyanidin dimer (type B)	2262.00 ± 63.00 a	332.50 ± 24.10 b	207.70 ± 112.10 b
42	Procyanidin trimer (type A)	9.27 ± 1.21 c	401.70 ± 2.00 a	231.40 ± 74.20 b
43	Procyanidin trimer (type B)	383.20 ± 14.90 a	104.60 ± 10.40 b	11.60 ± 1.65 c
44	Procyanidin tetramer	106.20 ± 4.18 a	16.00 ± 1.12 b	9.73 ± 2.91 b
45	Procyanidin pentamer	1.09 ± 0.06 a	0.67 ± 0.36 a	0.54 ± 0.12 a
46	Procyanidin hexamer	6.80 ± 0.02 a	3.01 ± 0.00 b	1.31 ± 0.14 c
47	Quercetin	0.69 ± 0.018 a	0.05 ± 0.01 c	0.39 ± 0.00 b
48	Quercetin arabinoside	0.70 ± 0.00 a	0.11 ± 0.01 c	0.61± 0.02 b
49	Quercetin glucoside	20.30 ± 0.75 a	3.95 ± 0.25 b	4.510 ± 0.11 b
50	Quercetin rhmanoside	2.07 ± 0.85	n.d	n.d.
51	Quercetin glucuronide	67.80 ± 0.18 a	0.08 ± 0.01 b	0.04 ± 0.00 b
52	Quercetin acetylglucoside	2.99 ± 0.09 a	0.17 ± 0.01 b	0.02 ± 0.01 c
53	Quercetin arabinoside glucoside	374.40 ± 22.70 a	0.68 ± 0.19 b	0.39 ± 0.08 b
54	Quercetin rutinoside	6.73 ± 0.19 a	0.36 ± 0.03 b	0.22 ± 0.10 b
55	Quercetin diglucoside	294.00 ± 1.35 a	4.51 ± 0.28 b	1.82 ± 0.17 c
56	Quercetin glucoside rhamnoside	4.98 ± 1.12	n.d	n.d.
57	Isorhamnetin	0.01 ± 0.00	n.d	n.d.
58	Isorhamnetin derivative	2.19 ± 0.08	n.d	n.d.
59	Isorhamnetin arabinoside	0.04 ± 0.00 c	0.98 ± 0.054 a	0.48 ± 0.12 b
60	Isorhamnetin glucoside	0.06 ± 0.00 b	0.15 ± 0.01 a	0.04 ± 0.01 b
61	Isorhamnetin glucuronide	9.20 ± 0.06 a	0.01 ± 0.00 b	n.d.
62	Isorhamnetin arabinoside glucoside	0.10 ± 0.00	n.d	n.d.
63	Kaempferol arabinoside	0.26 ± 0.00 a	n.d	0.09 ± 0.03 b
64	Kaempferol glucoside	4.81 ± 0.00 a	0.34 ± 0.04 c	0.64 ± 0.10 b
65	Kaempferol rutinoside	13.20 ± 0.06	n.d	n.d.
66	Kaempferol arabinoside glucoside	160.70 ± 0.00 a	0.28 ± 0.01 b	0.245 ± 0.02 b
67	Naringenin	0.27 ± 0.08 a	0.08 ± 0.00 b	0.19 ± 0.01 a b
68	Naringenin glucoside	0.59 ± 0.00 b	1.11 ± 0.08 a	0.00 ± 0.00 c
69	Sakuranetin	0.24 ± 0.00 b	n.d	0.86 ± 0.13 a
70	Luteolin	n.d.	0.02 ± 0.00 b	0.05 ± 0.01 a
71	Luteolin arabinoside glucoside	6.07 ± 0.33	n.d.	n.d.
	***Flavonoids***	***5721.86 ± 208.82 a***	***1826.99 ± 193.20 b***	***1135.68 ± 507.45 b***
72	Penstemide	2.82 ± 0.00 a	1.91 ± 0.06 b	0.62 ± 0.12 c
	***Terpenes***	***2.820 ± 0.00 a***	***1.91 ± 0.06 b***	***0.62 ± 0.12 c***
	**Total phenolic compounds**	**6836.35 ± 62.80 a**	**3850137 ± 356.09 b**	**1513.90 ± 578.20 c**

Data are expressed in µg of individual phenolic compound per 100 g as mean ± standard deviation (n = 3). Different letters indicate a statistical difference (*p* < 0.05) between the peel, seed coat, and seed extracts.

**Table 4:** Macronutrients and energy value of peel, seed coat and seed extracts from avocado fruit.

	Peel	Seed Coat	Seed
Carbohydrates (%)	36.39 ± 7.11 a	23.92 ± 1.23 b	28.13 ± 0.24 ab
Proteins (%)	2.82 ± 0.30 a	0.79 ± 0.01 b	0.69 ± 0.00 b
Fat (%)	7.68 ± 1.19 c	13.41 ± 0.82 b	21.55 ± 1.07 a
Moisture (%)	14.45 ± 0.26 a	15.14 ± 0.26 a	11.09 ± 0.33 b
Ash (%)	7.34 ± 0.34 a	6.46 ± 0.12 b	3.86 ± 0.03 c
Energy (kcal/100 g)	226.06 ± 0.06 b	219.62 ± 2.49 b	309.30 ± 8.65 a

Data are means ± standard deviation (n = 3). Different letters indicate a statistical difference (*p* < 0.05) between the peel, seed coat, and seed extracts.

**Table 5:** In-silico study of the phenolic compounds identified in the avocado peel, seed coat, and seed extracts.

	Identified Compound	MW	TPSA	Log P	No. Atoms	Hydrogen Bonds Acceptors	Hydrogen Bonds Donors	Rotatable Bonds	Molecular Volume (Å^3^)	Violations to LIRF	% ABS	log P_app_
1	*p*-Hydroxybenzoic acid	138.12	57.53	1.37	10	3	2	1	119.06	0	89.15	1.15
2	Vanillin	152.15	46.53	1.07	11	3	1	2	136.59	0	92.95	1.21
3	Vanillic acid	168.15	66.76	1.19	12	4	2	2	144.61	0	85.97	0.33
4	Syringic acid	198.17	76	1.20	14	5	2	3	170.15	0	82.78	0.49
5	Protocatechuic acid	154.12	77.75	0.88	11	4	3	1	127.08	0	82.18	0.49
6	Protocatechuic acid glucoside	302.24	156.91	−1.37	21	9	6	3	242.40	1	54.87	−0.66
7	Hydroxytyrosol	154.16	60.68	0.52	11	3	3	2	141.70	0	88.07	1.09
8	Hydroxytyrosol glucoside	316.31	139.84	−1.19	22	8	6	5	273.82	1	60.76	0.14
9	Hydroxysalidroside	316.31	139.84	−1.19	22	8	6	5	273.82	1	60.76	0.16
10	Hydroxytyrosol glucoside arabinoside	448.42	198.76	−2.78	31	12	8	7	381.10	2	40.43	−0.63
11	Tyrosol glucoside	300.31	119.61	−0.70	21	7	5	5	265.80	0	67.73	0.46
12	Salidroside	300.31	119.61	−0.70	21	7	5	5	265.80	0	67.73	0.46
13	Tyrosol glucoside arabinoside	432.42	178.53	−2.06	30	11	7	7	373.08	2	47.41	−0.44
14	*p*-coumaric acid	164.16	57.53	1.43	12	3	2	2	146.48	0	89.15	1.21
15	Coumaric acid glucoside	326.30	136.68	−0.36	23	8	5	5	278.60	0	61.85	−0.58
16	Coumaroyl quinic acid	338.31	144.52	0.28	24	8	5	5	288.60	0	59.14	0.80
17	Caffeic acid	180.16	77.75	0.94	13	4	3	2	154.50	0	82.18	0.63
18	Caffeic Acid Glucoside	342.30	156.91	−1.07	24	9	6	5	286.62	1	54.87	−0.67
19	Caffeic Acid Glucoside derivative	342.30	156.91	−1.07	24	9	6	5	286.62	1	54.87	−0.67
20	Dihydrocaffeic acid glucoside	330.29	156.91	−1.33	23	9	6	5	276.00	1	54.87	−0.08
21	Caffeoylshikimic acid	336.30	144.52	0.31	24	8	5	5	282.36	0	59.14	−0.59
22	3-*O*-caffeoylquinic acid	354.31	164.74	−0.45	25	9	6	5	296.27	1	52.16	−0.84
23	4-*O*-caffeoylquinic acid	354.31	164.74	−0.67	25	9	6	5	296.27	1	52.16	−0.89
24	5-*O*-caffeoylquinic acid	354.31	164.74	−0.45	25	9	6	5	296.27	1	52.16	−0.84
25	Dicaffeoylquinic acid	516.46	211.28	1.21	37	12	7	9	431.08	3	36.11	−1.20
26	Ferulic acid	194.19	66.76	1.25	14	4	2	3	172.03	0	85.97	0.17
27	Ferulic Acid Glucoside	356.33	145.91	−0.77	25	9	5	6	304.15	0	58.66	−0.55
28	Dihydroferulic acid glucoside	356.33	145.91	−0.77	25	9	5	6	304.15	0	58.66	−0.55
29	4-*O*-feruloylquinic acid	368.34	153.75	−0.36	26	9	5	6	313.80	0	55.96	−0.56
30	5-*O*-feruloylquinic acid	368.34	153.75	−0.14	26	9	5	6	313.80	0	55.96	−0.56
31	3-*O*-feruloylquinic acid	368.34	153.75	−0.14	26	9	5	6	313.80	0	55.96	−0.56
32	Catechin	290.27	110.37	1.37	21	6	5	1	244.14	0	70.92	−0.28
33	Epicatechin	290.27	110.37	1.37	21	6	5	1	244.14	0	70.92	−0.28
34	Catechin glucoside	452.41	189.53	−0.89	32	11	8	4	376.26	2	43.61	−0.88
35	epicatechin glucoside	452.41	189.53	−0.34	32	11	8	4	376.26	2	43.61	−0.92
36	Epigallocatechin	306.27	130.60	1.08	22	7	6	1	252.16	1	63.94	−0.37
37	Epicatechin gallate	442.38	177.13	2.54	32	10	7	4	359.55	1	47.89	−1.26
38	Catechin derivative	290.27	110.37	1.37	21	6	5	1	244.14	0	70.92	−0.28
39	Epicatechin derivative	290.27	110.37	1.37	21	6	5	1	244.14	0	70.92	−0.28
40	Procyanidin dimer (type A)	576.51	209.75	2.57	42	12	9	2	465.47	3	36.64	−1.08
41	Procyanidin dimer (type B)	578.53	220.75	2.14	42	12	10	3	475.67	3	32.84	−1.22
42	Procyanidin trimer (type A)	864.76	320.13	3.78	63	18	14	4	697.01	4	-1.44	−0.43
43	Procyanidin trimer (type B)	862.75	309.13	3.56	63	18	13	3	686.81	3	2.35	−1.82
44	Procyanidin tetramer	1157.05	441.50	4.13	84	24	20	7	944.98	3	-43.32	−2.62
45	Procyanidin pentamer	1443.29	551.87	6.22	105	30	25	9	1170.27	4	-81.40	−0.43
46	Procyanidin hexamer	1701.56	632.78	8.54	124	34	29	14	1395.59	4	-109.31	−0.36
47	Quercetin	302.24	131.35	1.68	22	11	7	1	240.08	0	63.68	−0.22
48	Quercetin arabinoside	434.35	190.28	0.06	31	11	7	3	347.36	2	43.35	0.15
49	Quercetin glucoside	464.38	210.50	−0.36	33	12	8	4	372.21	2	36.38	0.27
50	Quercetin rhamnoside	448.38	190.28	0.64	32	11	7	3	363.95	2	43.35	0.048
51	Quercetin glucuronide	478.36	227.57	−0.49	34	13	8	4	374.39	2	30.49	−1.06
52	Quercetin acetylglucoside	506.42	216.58	0.34	36	13	7	6	408.72	3	34.28	−0.00
53	Quercetin arabinoside glucoside	596.49	269.43	−1.73	42	16	10	6	479.48	3	16.05	−0.91
54	Quercetin rutinoside	610.52	269.43	−1.06	43	16	10	6	496.07	3	16.05	−1.59
55	Quercetin diglucoside	626.52	289.65	−2.38	44	17	11	7	504.33	3	9.07	−1.22
56	Quercetin glucoside rhamnoside	612.54	265.52	−2.00	43	16	10	6	502.31	3	17.40	−1.46
57	Isorhamnetin	316.26	120.36	1.99	23	7	4	0	257.61	0	67.48	−0.00
58	Isorhamnetin derivate	316.26	120.36	1.99	23	7	4	0	257.61	0	67.48	−0.00
59	Isorhamnetin arabinoside	448.38	179.28	0.37	32	11	6	2	364.89	2	47.15	0.37
60	Isorhamnetin glucoside	478.41	199.51	−0.06	34	12	7	5	389.73	2	40.17	0.33
61	Isorhamnetin glucoronide	492.39	216.58	−0.18	35	13	7	5	391.92	2	34.28	−0.85
62	Isorhamnetin arabinoside glucoside	610.52	258.43	−1.42	43	16	9	7	497.01	3	19.84	−0.99
63	Kaempferol arabinoside glycoside	580.50	249.20	−1.24	41	15	9	6	471.46	3	23.03	−0.40
64	Kaempferol glucoside	448.38	190.28	0.12	32	11	7	4	364.19	2	43.35	0.35
65	Kaempferol rutinoside	594.54	249.20	−0.57	42	15	9	6	488.05	3	23.03	0.18
66	Naringenin	272.26	86.99	2.12	20	5	3	1	230.26	0	78.99	1.02
67	Naringenin glucoside	434.40	177.13	−0.04	31	10	7	3	361.42	1	47.89	0.41
68	Sakuranetin	286.28	76.00	2.65	21	5	2	2	247.79	0	82.78	1.36
69	Luteolin	286.24	111.12	1.97	21	6	4	1	232.07	0	70.66	0.96
70	Luteolin arabinoside glucoside	610.52	291.42	−2.59	43	16	12	5	494.38	3	8,12	−1.40
71	Penstemide	296.36	76	1.76	21	5	2	6	282.65	0	82.78	1.09

MW = Molecular weight; Log P = octanol–water partition coefficient; Violations to LIRF = Violations to Lipinski’s rule of five; % ABS = Theoretical absorption percentage; log P_app_ = logarithm of the apparent permeability coefficient.

## Supplementary Materials

The following are available online at https://www.mdpi.com/article/10.3390/biom11070977/s1, Table S1: Commercial standards used for the phenolic compounds identification and quantification in the avocado peel, seed coat and seed extracts by UPLC-ESI-MS/MS, Table S2: Optimal selected reaction monitoring (SRM) conditions for the determination of phenolic compounds in avocado peel, seed coat and seed by UPLC-ESI-MS/MS.
