# Supplementary material for: Phenolic-Rich Extracts from Avocado Fruit Residues as Functional Food Ingredients with Antioxidant and Antiproliferative Properties
Source: Biomolecules. 2021 Jul 2;11(7):977. doi: 10.3390/biom11070977 (PMC8301936; doi:10.3390/biom11070977)
Supplement: Supplementary file 1 [file biomolecules-11-00977-s001.zip › biomolecules-1258223-supplementary.pdf]

Phenolic-Rich Extracts from Avocado Fruit Residues as Functional Food Ingredients with Antioxidant and Antiproliferative Properties

Gustavo R. Velderrain-Rodríguez [1], Javier Quero [2], Jesús Osada [2,3,4], Olga Martín-Belloso [1] and María Jesús Rodríguez-Yoldi [2,4,*]

[1] Agrotecnio Center, Department of Food Technology, University of Lleida, Av. Alcalde Rovira Roure 191, 25198 Lleida, Spain; grvelderrain@gmail.com (G.R.V.-R.); olga.martin@udl.cat (O.M.-B.)
[2] Department of Pharmacology and Physiology, Forensic and Legal Medicine, Veterinary Faculty, University of Zaragoza, 50013 Zaragoza, Spain; javierquero94@gmail.com (J.Q.); josada@unizar.es (J.O.)
[3] Department of Biochemistry and Molecular Cell Biology, Veterinary Faculty, University of Zaragoza, 50009 Zaragoza, Spain
[4] CIBERobn, ISCIII, IIS Aragón, IA2, 28029 Madrid, Spain
* Correspondence: mjrodyol@unizar.es; Tel.: +34-976-761649

SUPPLEMENTARY TABLES

Table S1. Commercial standards used for the phenolic compounds identification and quantification in the avocado peel, seed coat and seed extracts by UPLC-ESI-MS/MS.

	Compound	Standard Used for Quantification	Purchased From
1	p-Hydroxybenzoic Acid	p-Hydroxybenzoic Acid	Sigma-Aldrich
2	Vanillin	Vanillin	Sigma-Aldrich
3	Vanillic acid	Vanillic acid	Fluka
4	Syringic acid	Syringic acid	Sigma-Aldrich
5	Protocatehuic Acid	Protocatehuic Acid	Sigma-Aldrich
6	Protocatehuic Acid Glucoside	Protocatehuic Acid	
7	Hydroxytyrosol	Hydroxytyrosol	Extrasynthese
8	Hydroxytyrosol Glucoside	Hydroxytyrosol	
9	Hydroxysalidroside	Hydroxytyrosol	
10	Hydroxytyrosol Glucoside Arabinoside	Hydroxytyrosol	
11	Tyrosol Glucoside	Tyrosol	Sigma-aldrich
12	Salidroside	Tyrosol	
13	Tyrosol Glucoside Arabinoside	Tyrosol	
14	p-Cumaric Acid	p-Cumaric Acid	Extrasynthese
15	Coumaric acid glucoside	p-Cumaric Acid	
16	Coumaroylquinic Acid	p-Cumaric Acid	
17	Caffeic Acid	Caffeic Acid	Sigma-aldrich
18	Caffeic Acid Glucoside	Caffeic Acid	
19	Caffeic acid glucoside derivative	Caffeic acid	
20	Dihydrocaffeic acid glucoside	Caffeic acid	
21	Caffeoylshikimic Acid	Caffeic acid	
22	3-O-Caffeoylquinic Acid	5-O-Caffeoylquinic Acid	

23	4-O-Caffeoylquinic Acid	5-O-Caffeoylquinic Acid	Extrasynthese
24	5-O-Caffeoylquinic Acid	5-O-Caffeoylquinic Acid	
25	Dicaffeoylquinic acid	5-O-Caffeoylquinic Acid	
26	Ferulic Acid	Ferulic Acid	Fluka
27	Ferulic Acid Glucoside	Ferulic Acid	
28	Dihydroferulic Acid Glucoside	Ferulic Acid	
29	4-O-Feruloylquinic Acid	Ferulic Acid	
30	5-O-Feruloylquinic Acid	Ferulic Acid	
31	3-O-Feruloylquinic Acid	Ferulic Acid	
32	Catechin	Catechin	Sigma-Aldrich
33	Epicatechin	Epicatechin	Sigma-Aldrich
34	Catechin glucoside	Catechin	
35	Epicatechin Glucoside	Epicatechin	
36	Epigallocatechin	Epicatechin	
37	Epicatechin Gallate	Epicatechin	
38	Catechin derivative	Catechin	
39	Epicatechin derivative	Epicatechin	
40	Procyanidin dimer (type A)	Procyanidin dimer B2	Extrasynthese
41	Procyanidin dimer (type B)	Procyanidin dimer B2	
42	Procyanidin trimer (type A)	Procyanidin dimer B2	
43	Procyanidin trimer (type B)	Procyanidin dimer B2	
44	Procyanidin tetramer	Procyanidin dimer B2	
45	Procyanidin pentamer	Procyanidin dimer B2	
46	Procyanidin hexamer	Procyanidin hexamer	
47	Quercetin	Quercetin	Extrasynthese
48	Quercetin Arabinoside	Quercetin-3-O-glucoside	
49	Quercetin Glucoside	Quercetin-3-O-glucoside	Extrasynthese
50	Quercetin Rhamnoside	Quercetin-3-O-glucoside	
51	Quercetin Glucuronide	Quercetin	
52	Quercetin acetylglucoside	Quercetin	
53	Quercetin glucoside arabinoside	Quercetin-3-O-glucoside	
54	Quercetin Rutinoside	Quercetin Rutinoside	Extrasynthese
55	Quercetin Diglucoside	Quercetin-3-O-glucoside	
56	Quercetin glucoside rhamnoside	Quercetin-3-O-glucoside	
57	Isorhamnetin	Isorhamnetin	Extrasynthese
58	Isorhamnetin Derivate	Isorhamnetin	
59	Isorhamnetin Arabinoside	Isorhamnetin	
60	Isorhamnetin glucoside	Isorhamnetin	
61	Isorhamnetin Glucuronide	Isorhamnetin	
62	Isorhamnetin Arabinoside Glucoside	Isorhamnetin	
63	Kaempferol arabinoside	Kaempferol-3-O-glucoside	Extrasynthese
64	Kaempferol Glucoside	Kaempferol-3-O-glucoside	
65	Kaempferol Rutinoside	Kaempferol-3-O-glucoside	
66	Kaempferol Arabinoside Glucoside	Kaempferol-3-O-glucoside	
67	Naringenin	Naringenin	Extrasynthese
68	Naringenin glucoside	Naringenin	
69	Sakuratetin	Naringenin	
70	Luteolin	Luteolin	Extrasynthese
71	Luteolin arabinoside glucoside	Luteolin	
72	Penstemide	Quercetin	

Table S2. Optimal selected reaction monitoring (SRM) conditions for the determination of phenolic compounds in avocado peel, seed coat and seed by UPLC-ESI-MS/MS.

	Compound	MW (g/mol)	SRM (Quantification)	Cone Voltage (V)	Collision Energy (eV)	Detected in
1	p-Hydroxybenzoic Acid	138	137 > 93	30	15	Peel, Seed coat and Seed
2	Vanillin	152	151 > 136	25	10	Peel, Seed coat and Seed
3	Vanillic acid	168	167 > 123	30	10	Peel
4	Syringic acid	198	197 > 182	30	10	Peel
5	Protocatehuic Acid	154	153 > 109	40	15	Peel and seed
6	Protocatehuic Acid Glucoside	316	315 > 153	40	20	Peel, Seed coat and Seed
7	Hydroxytyrosol	154	153 > 123	35	10	Peel, Seed coat and Seed
8	Hydroxytyrosol Glucoside	316	315 > 153	40	20	Peel, Seed coat and Seed
9	Hydroxysalidroside	316	315 > 135	40	30	Peel, Seed coat and Seed
10	Hydroxytyrosol Glucoside Arabinoside	448	447 > 153	40	20	Peel and Seed coat
11	Tyrosol Glucoside	300	299 > 137	40	20	Peel, Seed coat and Seed
12	Salidroside	300	299 > 179	40	10	Peel, Seed coat and Seed
13	Tyrosol Glucoside Arabinoside	432	431 > 137	40	20	Peel, Seed coat and Seed
14	p-Cumaric Acid	164	163 > 119	35	10	Peel, Seed coat and Seed
15	Coumaric acid glucoside	326	325 > 163	40	20	Peel
16	Coumaroylquinic Acid	338	337 > 191	40	20	Peel, Seed coat and Seed
17	Caffeic Acid	180	179 > 135	35	15	Peel, Seed coat and Seed

18	Caffeic Acid Glucoside	342	341 > 179	40	20	Peel, Seed coat and Seed
19	Caffeic acid glucoside derivative	546	545 > 341	40	20	Peel
20	Dihydrocaffeic acid glucoside	344	343 > 181	40	20	Peel, Seed coat and Seed
21	Caffeoylshikimic Acid	336	335 > 161	40	20	Peel, Seed coat and Seed
22	3-O-Caffeoylquinic Acid	354	353 > 179	40	15	Peel, Seed coat and Seed
23	4-O-Caffeoylquinic Acid	354	353 > 173	40	15	Peel, Seed coat and Seed
24	5-O-Caffeoylquinic Acid	354	353 > 191	40	15	Peel, Seed coat and Seed
25	Dicaffeoylquinic acid	516	515 > 191	40	30	Peel
26	Ferulic Acid	194	193 > 134	30	15	Peel, Seed coat and Seed
27	Ferulic Acid Glucoside	356	355 > 193	40	20	Peel, Seed coat and Seed
28	Dihydroferulic Acid Glucoside	358	357 > 195	40	20	Peel, Seed coat and Seed
29	4-O-Feruloylquinic Acid	368	367 > 173	40	20	Peel, Seed coat and Seed
30	5-O-Feruloylquinic Acid	368	367 > 191	40	15	Peel, Seed coat and Seed
31	3-O-Feruloylquinic Acid	368	367 > 193	40	15	Peel, Seed coat and Seed
32	Catechin	290	289 > 245	40	15	Seed coat and Seed
33	Epicatechin	290	289 > 245	40	15	Peel, Seed coat and Seed
34	Catechin glucoside	452	451 > 289	40	25	Peel, Seed coat and Seed

35	Epicatechin Glucoside	452	451 > 289	40	25	Peel, Seed coat and Seed
36	Epigallocatechin	306	305 > 125	40	15	Peel, Seed coat and Seed
37	Epicatechin Gallate	442	441 > 169	40	20	Seed coat and Seed
38	Catechin derivative	740	739 > 289	40	30	Peel, Seed coat and Seed
39	Epicatechin derivative	740	739 > 289	40	30	Peel, Seed coat and Seed
40	Procyanidin dimer (type A)	576	575 > 285	40	20	Peel, Seed coat and Seed
41	Procyanidin dimer (type B)	578	577 > 289	40	20	Peel, Seed coat and Seed
42	Procyanidin trimer (type A)	864	863 > 411	40	30	Peel, Seed coat and Seed
43	Procyanidin trimer (type B)	866	865 > 287	60	30	Peel, Seed coat and Seed
44	Procyanidin tetramer	1154	1153 > 865	70	20	Peel, Seed coat and Seed
45	Procyanidin pentamer	1442	1441 > 1028	80	25	Peel, Seed coat and Seed
46	Procyanidin hexamer	1730	1729 > 1153	80	30	Peel, Seed coat and Seed
47	Quercetin	302	301 > 151	40	15	Peel, Seed coat and Seed
48	Quercetin Arabinoside	434	433 > 300	40	20	Peel, Seed coat and Seed
49	Quercetin Glucoside	464	463 > 300	40	30	Peel, Seed coat and Seed
50	Quercetin Rhamnoside	478	477 > 301	40	25	Peel
51	Quercetin Glucuronide	478	477 > 301	40	25	Peel, Seed coat and Seed

52	Quercetin acetylglucoside	506	505 > 300	40	25	Peel, Seed coat and Seed
53	Quercetin glucoside arabinoside	596	595 > 300	40	30	Peel, Seed coat and Seed
54	Quercetin Rutinoside	610	609 > 300	40	30	Peel, Seed coat and Seed
55	Quercetin Diglucoside	626	625 > 300	40	30	Peel, Seed coat and Seed
56	Quercetin glucoside rhamnoside	756	755 > 300	40	35	Peel
57	Isorhamnetin	316	315 > 300	40	15	Peel
58	Isorhamnetin Derivate		300 > 315	40	15	Peel
59	Isorhamnetin Arabinoside	448	447 > 315	40	20	Peel, Seed coat and Seed
60	Isorhamnetin glucoside	478	477 > 315	40	20	Peel, Seed coat and Seed
61	Isorhamnetin Glucuronide	492	491 > 315	40	20	Peel and Seed coat
62	Isorhamnetin Arabinoside Glucoside	610	609 > 315	40	30	Peel
63	Kaempferol arabinoside	418	417 > 284	40	20	Peel and Seed
64	Kaempferol Glucoside	448	447 > 284	40	20	Peel, Seed coat and Seed
65	Kaempferol Rutinoside	594	593 > 284	40	30	Peel
66	Kaempferol Arabinoside Glucoside	580	579 > 284	40	30	Peel, Seed coat and Seed
67	Naringenin	272	271 > 151	40	15	Peel, Seed coat and Seed
68	Naringenin glucoside	434	433 > 271	40	20	Peel, Seed coat and Seed
69	Sakuratetin	286	285 > 199	40	20	Peel and Seed
70	Luteolin	286	285 > 133	40	20	Seed and Seed

71	Luteolin arabinoside glucoside	580	579 > 285	40	30	Peel
72	Penstemide	444	443 > 119	40	25	Peel, Seed coat and Seed
